# Synergistic Effects of Thread Lifting, Autologous Fat Grafting, and Multi-Modal Laser Therapy in Facial Rejuvenation

**DOI:** 10.1093/asjof/ojaf139

**Published:** 2025-10-28

**Authors:** Naci Celik

## Abstract

**Background:**

Facial aging is a multifactorial process involving tissue descent, volume depletion, and dermal degeneration. Minimally invasive modalities like thread lifting, autologous fat grafting, and laser treatments address different aging components. However, their synergistic impact remains underexplored.

**Objectives:**

The aim of the author of this study is to assess the clinical efficacy and histologic rationale of combining permanent thread lifting, autologous fat grafting, and nonablative laser therapy for comprehensive facial rejuvenation.

**Methods:**

In this retrospective study, a total of 65 female patients (aged 31-60, Fitzpatrick skin types II-IV) treated from January 2022 to January 2023 were included. Patients were divided into 3 groups: thread lifting alone (Group 1), thread + laser (Group 2), and thread + laser + fat grafting (Group 3). Evaluations were performed at 12 months by a plastic surgeon, a dermatologist, and the patients themselves using a 5-point Likert-scale assessing skin texture, fine wrinkles, lift effect, and overall satisfaction. Kruskal–Wallis and Mann–Whitney *U* tests with Bonferroni correction were utilized, alongside cumulative link regression models.

**Results:**

Significant group differences were observed in skin texture (*P* = .00013) and overall satisfaction (*P* = .00421). Post hoc comparisons revealed that Groups 2 and 3 outperformed Group 1 in skin texture (*P* = .0155 and *P* = .0003, respectively). Fine wrinkle improvement was notable in Group 3 (*P* = .0239 vs Group 1). Ordinal logistic regression confirmed significant improvement with laser therapy per dermatologist ratings (odds ratio = 2.10, *P* = .019), with a favorable trend observed for fat grafting. Cliff's δ and Hodges–Lehmann estimates showed progressively larger improvements with increasing treatment complexity.

**Conclusions:**

Combining thread lifting, autologous fat grafting, and laser therapy may provide superior aesthetic outcomes to thread lifting ± laser therapy alone. This is likely the result of the synergistic effects on mechanical lift, volume restoration, and dermal remodeling. These findings support an integrative treatment paradigm for nonsurgical facial rejuvenation and justify further prospective validation with histologic endpoints.

**Level of Evidence: 3 (Therapeutic):**

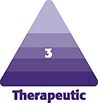

Facial aging is a multifactorial and dynamic process characterized by progressive changes in skin quality, soft tissue volume, fat distribution, and underlying skeletal structure. As early as the 20th century, surgical rhytidectomy was the primary tool for facial rejuvenation, focusing primarily on the mechanical repositioning of sagging tissues. However, this unidimensional approach failed to address cutaneous aging or volume depletion. Over time, it became clear that aging is not only a gravitational problem but also involves cellular and extracellular matrix (ECM) degeneration, loss of elasticity, pigmentary disorders, and volume atrophy.

The aesthetic field evolved to include multiple modalities targeting different aging mechanisms. Thread lifting, first conceptualized in the mid-1990s, gained popularity with the introduction of barbed sutures that allowed for mechanical suspension without traditional incisions. Dr Marlen Sulamanidze pioneered the APTOS thread in 1999, which laid the groundwork for later innovations in permanent and resorbable suspension systems.^1^ When anchored correctly in the superficial musculoaponeurotic system (SMAS), permanent silicone or polypropylene threads may provide predictable and long-lasting lift. Modern techniques emphasize vector-based elevation and are widely utilized in patients seeking minimally invasive alternatives to surgical facelifts.^2^

Autologous fat grafting has a long history dating back to the late 19th century when German surgeon Gustav Neuber described fat transfer for scar correction in 1893. However, the modern resurgence of fat grafting in aesthetic medicine occurred in the 1990s, championed by Dr Coleman, who developed a standardized fat harvesting, processing, and reinjection technique. Alternative techniques like Brava (Brava LLC, Miami, FL)-assisted fat transfer, pioneered by Khouri et al, have demonstrated safety and efficacy in volumization and tissue expansion contexts.^3,4^ Today, fat grafting is recognized not only for its volumizing effects but also for its regenerative potential, attributed to the presence of adipose-derived stem cells (ADSCs) and the stromal vascular fraction (SVF).^5-7^ These elements stimulate neocollagenesis, angiogenesis, and dermal remodeling, providing benefits beyond volume restoration. Yoshimura et al's work on cell-assisted fat transfer has shown that enrichment with ADSCs significantly improves graft survival and regenerative response.^8,9^

Laser therapy for skin rejuvenation has evolved significantly since the first clinical use of CO_2_ lasers in the 1980s. Although effective, early ablative lasers were associated with considerable downtime and adverse effects. The Er:YAG laser, introduced in the 1990s, offered a safer alternative with its precise ablation limited to the epidermis and upper dermis. In a special SMOOTH mode, Er:YAG operates nonablatively to induce controlled thermal injury, stimulating collagen production with minimal surface disruption (<5 µm).^10^ Nd:YAG lasers emerged later as deep-penetrating tools for bulk dermis heating, encouraging elastin fiber reorganization and tightening without damaging the epidermis.^11^ Q-switched Nd:YAG lasers were initially developed for tattoo and pigment removal, targeting melanin chromophores through selective photothermolysis. With pulse durations in the nanosecond range, these lasers fragment pigmented lesions while sparing surrounding tissues. Their role in treating melasma, lentigines, and other dyschromias has been well-established, making them valuable adjuncts in comprehensive facial rejuvenation.^12^

Given the strengths and limitations of each modality, the modern approach to facial rejuvenation is increasingly integrative. Combining thread lifting for mechanical repositioning, fat grafting for volume and regeneration, and laser therapies for surface renewal and pigment correction allows practitioners to treat aging as a composite degenerative condition. In the present study, the author evaluates the outcomes of such a tri-modal strategy, analyzing clinical improvements and reviewing the supporting histologic mechanisms described in the literature.

## METHODS

This was a retrospective cohort analysis of consecutive female patients treated at a private clinic between January 2022 and January 2023. No age cutoffs were applied other than adult status (≥18 years).

The inclusion criteria were healthy female patients without systemic diseases who opted for facial rejuvenation procedures and completed a minimum follow-up period of 12 months.

Exclusion criteria aimed to standardize the patient population and reduce variables potentially affecting study outcomes. Patients excluded were those who underwent simultaneous aesthetic surgical procedures (eg, facelift, blepharoplasty, and rhinoplasty), individuals receiving filler injections or botulinum toxin treatments within the follow-up period, smokers, and those with any facial surgery within 12 months before or after the studied intervention. Additionally, male patients were excluded because of differing anatomical characteristics, such as facial hair density, which could significantly alter laser treatment protocols and introduce outcome variability. Furthermore, international patients and those who underwent concurrent nonfacial aesthetic or surgical procedures were also excluded to maintain uniformity in patient demographics and treatment protocols.

Allocation into treatment groups was patient driven rather than predetermined. Patients were informed about the standard clinical recommendation (thread lifting + laser therapy + autologous fat grafting). However, final treatment selection reflected patient preferences and financial considerations. As a result, some elected to undergo thread lifting alone or thread lifting with laser therapy only.

### Ethical Approval and Consent

This study was approved by the Non-Interventional Research Ethics Committee (Ref: 61351342/Jan 2021-07). Written informed consent for treatment and publication of clinical data and photographs was obtained from all participants.

### Thread Operation (All Groups)

In all patients, midface and lower face lifting was performed using nonabsorbable silicone suspension threads (Infinite Thread, Thread & Lift, France). For midface elevation, threads were inserted through temporal stab incisions; for lower face and neck repositioning, entry was made through a point inferior to the earlobe. When indicated, submental liposuction was performed using a Tonnard cannula and a 10 mL Luer-lock syringe to optimize cervical contour. Thread vectors followed a deep subcutaneous plane, targeting the SMAS to achieve vertical and oblique lifting. The detailed technique, including vector planning, anchoring, and 3-dimensional correction of tissue ptosis, has been previously described in the literature.^13^

### Laser Treatments (Groups 2 and 3)

Laser treatments were performed using a combination of Fotona SP Dynamis (Fotona, Ljubljana, Slovenia; Er:YAG and Nd:YAG) and StarWalker (Fotona; Q-Switched Nd:YAG) laser systems. Treatments commenced 1 week after surgery for patients in Groups 2 and 3. Each patient received 4 treatment sessions: Sessions 1 through 3 were spaced 1 month apart, with the final (fourth) session conducted 3 months after the third session. No anesthesia or topical anesthetic creams were employed during these laser sessions.

Each laser session adhered to the following standardized, stepwise protocol:

Step 1: Er:YAG (SMOOTH Mode): 625 ms pulse duration, 5 stacks at 10.50 J/cm^2^ fluence, single-pass coverage of the entire face and neck.
Step 2: Nd:YAG (PIANO Mode): 140 J/cm^2^ fluence, 9 mm spot size, and pulse duration set to 5 s per pulse, without cooling. Target skin temperature was continuously maintained at 39°C to 41°C for 5 min per side on each cheek and lower facial area.
Step 3: Nd:YAG (FRAC3 Mode): 1.6 ms pulse duration, 25 J/cm^2^ fluence, 4 Hz frequency, 4 passes covering the entire face. The integrated cooling system was active during this step.
Step 4: Q-Switched Nd:YAG (StarWalker): 1.8 J/cm^2^ fluence, 8 mm lens size, 6 Hz frequency, 6 passes covering the entire face.

### Autologous Fat Harvesting and Injection (Group 3 Only)

Fat harvesting and thread lifting were performed during the same operative session, and autologous fat grafting was performed immediately before thread placement. Fat was harvested manually from the lower abdomen under sterile conditions using a Tonnard cannula and a 10 mL Luer-lock syringe. The lipoaspirate was centrifuged at 3000 rpm for 3 min to separate oil, fluid, and purified fat layers. Fat grafting was performed with 1 mL Luer-lock syringes using a 19-G disposable blunt cannula, employing a multilayered microdroplet technique across the subcutaneous and deep compartments.

### Outcome Assessment

Aesthetic outcomes were evaluated 12 months posttreatment through standardized photographic documentation. Three evaluators—a plastic surgeon, a dermatologist, and the patient (self-assessment)—independently rated improvement using a 5-point Likert scale adapted from validated aesthetic outcome measures and patient satisfaction scores previously reported in thread-lifting studies.^14^ The plastic surgeon and dermatologist performed their assessments in a blinded fashion. Parameters included skin texture, pigmentation, fine wrinkles, lifting effect, and overall aesthetic enhancement. Inter-rater reliability between the 2 blinded evaluators was assessed using Cohen's kappa statistic.

### Statistical Analysis

Outcomes were assessed for skin texture, fine wrinkles, lift effect, and overall satisfaction. Group comparisons were performed using the Kruskal–Wallis test. When significant differences were detected, pairwise comparisons were conducted with the Mann–Whitney *U* test, applying Bonferroni correction. To evaluate the influence of covariates such as patient age, number of threads utilized, and fat graft volume, multivariable ordinal logistic regression analyses were performed separately for each evaluator. Nonparametric tests were selected because Likert-scale data may not meet parametric assumptions. Bonferroni correction provided robust control over false positives in post hoc testing. Ordinal regression modeling allowed for the appropriate handling of ordered categorical data and provided adjusted effect estimates.

All analyses were performed in Python 3.11.8 (Python Software Foundation). SciPy 1.9.3 was utilized for nonparametric tests, including Kruskal–Wallis and Mann–Whitney *U*. Stats models 0.13.5 was employed for cumulative link (ordinal logistic) regression and multiple-testing correction. Data wrangling and descriptive statistics were performed using pandas 1.5.3 and NumPy 1.25.x.

## RESULTS

A total of 65 female patients were included. The mean age was 37.5 ± 5.6 years (range, 31-60 years). Fitzpatrick skin types ranged from II to IV, with a predominance of Types III and IV. Baseline demographic and clinical characteristics are summarized in Table 1. There were no statistically significant differences among the 3 groups regarding age (*P* = .12) or Fitzpatrick skin type distribution (*P* = .97). Submental liposuction was performed in 6 patients (26.1%) in Group 1, 7 patients (28.0%) in Group 2, and 5 patients (29.4%) in Group 3, with comparable proportions across groups (*P* = .94). In Group 3, autologous fat grafting was performed with a total volume of 12 to 20 mL per patient (mean 16.0 ± 2.4 mL).

**Table 1.** Baseline Demographic and Clinical Characteristics of the Study Groups

Characteristic	Group 1 (thread only, *n* = 23)	Group 2 (thread + laser, *n* = 25)	Group 3 (thread + laser + fat, *n* = 17)	*P*-value
Age, years (mean ± SD)	38.7 ± 5.4	35.4 ± 3.4	38.9 ± 7.4	.12
Fitzpatrick II, *n* (%)	5 (21.7)	5 (20.0)	4 (23.5)	
Fitzpatrick III, *n* (%)	10 (43.5)	11 (44.0)	7 (41.2)	
Fitzpatrick IV, *n* (%)	8 (34.8)	9 (36.0)	6 (35.3)	
Overall Fitzpatrick distribution				.97
Submental liposuction, *n* (%)	6 (26.1)	7 (28.0)	5 (29.4)	.94

No significant differences were observed for age (*P* = .12), Fitzpatrick skin type distribution (*P* = .97), or submental liposuction rates (*P* = .94). Data are mean ± SD or *n* (%). P-values from Kruskal–Wallis (continuous variables) and chi-square (categorical variables). SD, standard deviation.

Baseline aesthetic evaluation is shown in Table 2. No statistically significant differences were found between groups regarding skin texture or fine wrinkles (surgeon: skin texture *P* = .1512, fine wrinkles *P* = .1860; dermatologist: skin texture *P* = .0921, fine wrinkles *P* = .4773). Pigmentation scores assessed by the dermatologist yielded a statistically significant difference (*P* = .0257), whereas surgeon ratings for pigmentation showed no significant difference (*P* = .0730). The inter-rater agreement between evaluators was low (*κ* = −0.01 for skin texture, *κ* = 0.02 for fine wrinkles, and *κ* = 0.12 for pigmentation), reflecting limited concordance between the plastic surgeon and dermatologist.

**Table 2.** Baseline Aesthetic Evaluation by Blinded Reviewers (Plastic Surgeon and Dermatologist)

Parameter	Evaluator	Group 1 (thread only, *n* = 23)	Group 2 (thread + laser, *n* = 25)	Group 3 (thread + laser + fat, *n* = 17)	*P*-value
Skin Texture	Surgeon	3.09 ± 0.67	3.00 ± 0.65	2.71 ± 0.47	.1512
Skin Texture	Dermatologist	3.22 ± 0.67	2.84 ± 0.55	3.12 ± 0.60	.0921
Fine Wrinkles	Surgeon	2.83 ± 0.78	3.24 ± 0.72	3.06 ± 0.56	.1860
Fine Wrinkles	Dermatologist	3.35 ± 0.78	3.24 ± 0.72	3.06 ± 0.83	.4773
Pigmentation	Surgeon	2.70 ± 0.76	2.88 ± 0.33	3.12 ± 0.60	.0730
Pigmentation	Dermatologist	2.48 ± 0.59	2.92 ± 0.49	2.76 ± 0.66	.0257*

No significant differences were observed in skin texture or fine wrinkles across groups. Dermatologist-assessed pigmentation showed a significant difference (*P* = .0257), whereas surgeon-assessed pigmentation did not (*P* = .0730). Values are mean ± SD, based on a 5-point Likert scale (1 = none, 5 = severe). Kruskal–Wallis test used for between-group comparisons. SD, standard deviation. *, statistically significant difference (*P* < 0.05)

The number of threads varied slightly between groups (mean 5.0 ± 0.9 in Group 1, 4.5 ± 1.0 in Group 2, and 4.9 ± 0.8 in Group 3), with no statistically significant difference observed (Kruskal–Wallis *P* = .1410).

**Figure 1.**
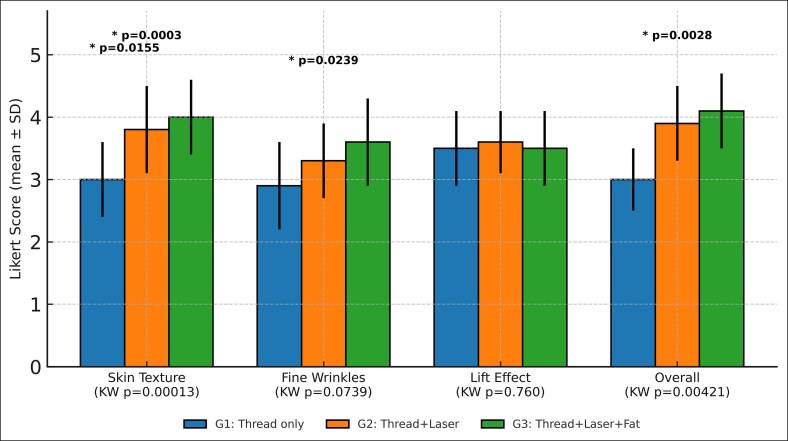
Aesthetic outcomes at 12 months by treatment group. Mean Likert scores (±standard deviation) are shown for skin texture, fine wrinkles, lift effect, and overall outcome (1 = no improvement, 5 = excellent). Groups: G1 = thread only; G2 = thread + laser; G3 = thread + laser + fat. Kruskal–Wallis (KW) global test *P*-values are displayed below the x-axis to indicate overall group differences. Post hoc pairwise comparisons (Bonferroni-adjusted Mann–Whitney *U* tests) are displayed above the bars. Multiple *P*-values are shown where >1 pairwise comparison reached statistical significance (eg, skin texture, where both Group 1 vs Group 2 and Group 1 vs Group 3 were significant).

Aesthetic outcomes at 12 months are illustrated in Figure 1. The Kruskal–Wallis test demonstrated significant differences in skin texture (*P* = .00013) and overall satisfaction (*P* = .00421). Differences for fine wrinkles approached significance (*P* = .0739), whereas lift effect ratings were similar across groups (*P* = .760). Post hoc pairwise comparisons further revealed that Groups 2 and 3 had significantly greater improvements in skin texture compared with Group 1 (*P* = .0155 and *P* = .0003, respectively). For fine wrinkles, a significant difference was observed between Groups 1 and 3 (*P* = .0239). Overall aesthetic improvement was significantly greater in Group 3 compared with Group 1 (*P* = .0028). No significant differences were observed between Groups 2 and 3.

**Figure 2.**
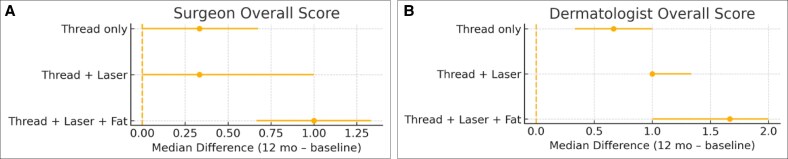
Composite forest plots of overall aesthetic improvement at 12 months. Paired median changes from baseline are shown with 95% bootstrap CIs. (A) Plastic surgeon overall scores. (B) Dermatologist overall scores. The vertical dashed line represents no change. Both evaluators demonstrated consistent patterns of improvement, with dermatologist ratings showing greater magnitudes of change compared with surgeon ratings.

Median paired improvements with 95% CIs are illustrated in Figure 2. Panel A shows plastic surgeon ratings, and Panel B shows dermatologist ratings. Both evaluators demonstrated consistent patterns of improvement across domains, with the dermatologist reporting higher scores for skin texture and pigmentation.

Ordinal logistic regression analysis (Table 3) confirmed a statistically significant improvement in dermatologist ratings for Group 2 vs Group 1 (odds ratio [OR] = 2.10, 95% CI, 1.12-4.02, *P* = .019). A strong but nonsignificant trend was observed for Group 3 vs Group 1 (OR = 3.84, 95% CI, 0.91-16.2, *P* = .072). Evaluations by patients and the plastic surgeon did not reach statistical significance.

**Table 3.** Ordinal Logistic Regression Analysis of Overall Aesthetic Outcomes.

Evaluator	Group 2 vs 1 OR (95% CI)	*P*-value	Group 3 vs 1 OR (95% CI)	*P*-value	Pseudo R²
Patient	1.88 (0.98-3.65)	.058	1.05 (0.52-2.13)	.89	0.181
Plastic Surgeon	1.42 (0.75-2.69)	.28	2.51 (0.93-6.81)	.072	0.131
Dermatologist	2.10 (1.12-4.02)	.019*	3.84 (0.91-16.2)	.072	0.287

A significant improvement was identified only in dermatologist ratings for Group 2 (thread + laser) versus Group 1 (thread only) (OR = 2.10, 95% CI, 1.12-4.02, *P* = .019). *, statistically significant difference (*P* < 0.05). Group 3 versus Group 1 showed a nonsignificant trend (OR = 3.84, *P* = .072). Patient and surgeon ratings did not reach statistical significance. OR, odds ratio; CI, confidence interval.

Effect size estimates (Table 4) demonstrated larger improvements in Groups 2 and 3 compared with Group 1. Dermatologist evaluations yielded the largest effect sizes, particularly for Group 3 (Cliff's *δ* = 0.82; median improvement +2.1).

**Table 4.** Effect Sizes for Aesthetic Improvement at 12 Months Compared With Baseline

Group	n (pairs)	Cliff's δ (Surgeon)	Cliff's δ (Dermatologist)	Median Difference (Surgeon)	Median Difference (Dermatologist)
Thread Only	23	0.34 (small)	0.48 (medium)	+0.5	+0.6
Thread + Laser	25	0.42 (medium)	0.66 (large)	+1.0	+1.4
Thread + Laser + Fat	17	0.59 (large)	0.82 (very large)	+1.5	+2.1

Effect sizes increased progressively with treatment complexity. Surgeon evaluations showed a small effect for thread Only (*δ* = 0.34), a medium effect for thread + laser (*δ* = 0.42), and a large effect for thread + laser + fat (δ = 0.59). Dermatologist evaluations demonstrated higher magnitudes overall, with a very large effect in the thread + laser + fat group (*δ* = 0.82; Hodges–Lehmann median improvement +2.1). Effect sizes were estimated using Cliff's δ and Hodges–Lehmann Median Differences. δ = Cliff's delta.

### Complications

Surgical complications were minor and manageable. Mild asymmetry occurred in 1 patient from Group 1, corrected with an additional pair of threads placed under local anesthesia 2 weeks postoperatively. Transient edema and erythema occurred universally in patients who underwent laser therapy, typically resolving within 1 to 3 days. Minor skin dimplings at thread entry/exit points were noted and effectively resolved with manual massage within 2 to 7 days postoperatively. No foreign body reactions, infections, or thread extrusions were observed in any patient.

### Representative Clinical Cases

**Figure 3.**
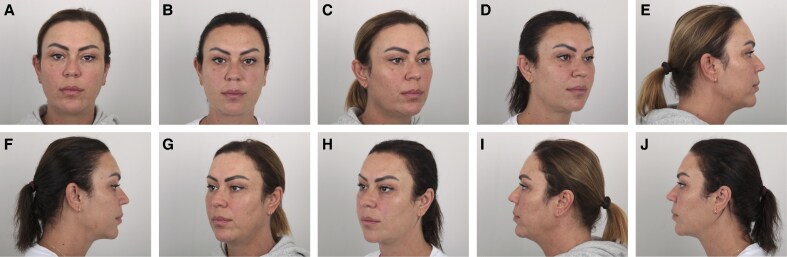
Standardized clinical photographs of a 41-year-old female patient who underwent facial thread lifting with permanent silicone suspension threads (Group 1). A total of 5 thread pairs were placed. Concomitant submental liposuction was performed with 25 cc of lipoaspirate removed. (A, C, E, G, I) Preoperative views. (B, D, F, H, J) 18-month postoperative views.

**Figure 4.**
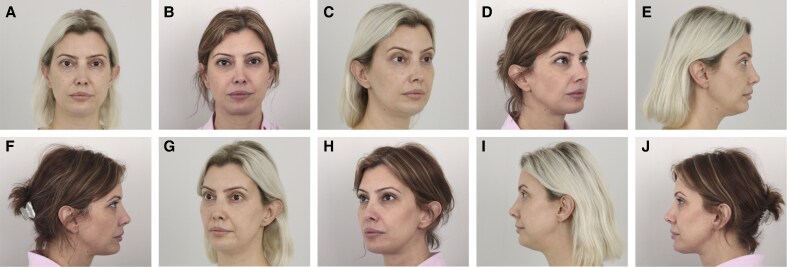
Standardized clinical photographs of a 40-year-old female patient who underwent facial thread lifting with permanent silicone suspension threads and 4 sessions of multimodal laser therapy (Group 2). A total of 5 thread pairs were placed. Concomitant submental liposuction was performed with 15 cc of lipoaspirate removed. (A, C, E, G, I) Preoperative views. (B, D, F, H, J) Eighteenth-month postoperative views.

**Figure 5.**
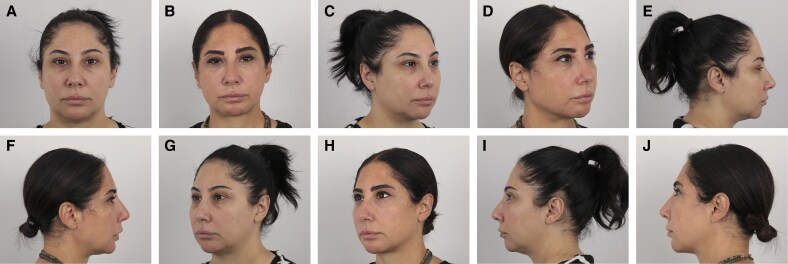
Standardized clinical photographs of a 41-year-old female patient who underwent facial thread lifting with permanent silicone suspension threads, autologous fat grafting, and 4 sessions of multimodal laser therapy (Group 3). A total of 5 thread pairs were placed. Sixteen milliliters of autologous fat were injected into the jawline, mentum, and infraorbital region. Concomitant submental liposuction was performed, yielding 20 cc of lipoaspirate. (A, C, E, G, I). Preoperative views. (B, D, F, H, J) Twentieth-month postoperative views.

Representative results from 1 patient in each treatment group are shown in Figures 3-5. These cases illustrate the progressive aesthetic improvement observed with increasing treatment complexity.

## DISCUSSION

Thread lifting with permanent silicone or polypropylene suspension threads remains a cornerstone of minimally invasive facial rejuvenation. It achieves immediate mechanical lifting by anchorage within the SMAS, with subsequent stabilization occurring through localized fibrosis. Histological evidence demonstrates fibroblast proliferation, collagen realignment, and dermal thickening along the thread tracks.^15,16^ Although threads effectively address mechanical tissue descent with consistent outcomes and low complication rates, they do not significantly enhance skin quality parameters such as pigmentation, hydration, or fine textural improvements.

Recent literature underscores that using permanent suspension sutures remains debated among plastic surgeons, mainly because of concerns about potential late complications such as extrusion, palpability, or foreign body reactions.^17,18^ Nevertheless, long-term data—although limited—indicate a favorable safety profile when correct technique and patient selection are applied. The largest prospective series to date by Few et al reported high patient satisfaction and durable lifting results over extended follow-up in 100 absorbable suture suspension cases.^19^ More recently, Hügül et al provided objective ultrasonographic evidence of the long-term stability of permanent silicone suspension sutures.^20^ In their cohort of 51 patients followed for up to 91 months after thread lifting with Infinite Thread or Spring Thread, no migration, extrusion, or significant degradation was observed. Mean thread thickness remained stable beyond the first 6 months postprocedure, and threads were still clearly visible on ultrasound after 7 years. These findings suggest preserved mechanical integrity and biocompatibility over extended periods, likely reinforced by perithread fibrosis and encapsulation, which may help maintain lift and prevent displacement. Such objective imaging data complement earlier long-term observational studies based on photographic and patient-reported outcomes, adding robust evidence to the safety profile of permanent suture suspension.

Autologous fat grafting adds a regenerative dimension by leveraging the SVF, which contains ADSCs, pericytes, endothelial progenitors, and immunomodulatory macrophages.^21^ Zuk et al initially characterized ADSCs as multipotent progenitors capable of mesenchymal differentiation, whereas Rigotti et al demonstrated their clinical efficacy in irradiated tissues.^22,23^ Fat grafts stimulate neocollagenesis, ECM remodeling, and epidermal repair, enhancing angiogenesis and dermal hydration through cytokines and growth factor secretion.^24-26^ Additionally, fat grafts modulate inflammation by promoting M2 macrophage polarization and suppressing pro-inflammatory cytokines.^27,28^ Emerging evidence also suggests that these regenerative effects are potentiated when combined with photothermal modalities such as laser therapy.^29^

Laser therapy complements these interventions through distinct photothermal mechanisms. Er:YAG lasers at 2940 nm target water, providing superficial thermal coagulation in SMOOTH mode, which promotes collagen remodeling and heat shock protein (HSP) expression.^30-33^ Histologically, this results in epidermal thickening and enhanced dermal–epidermal junction integrity. Nd:YAG lasers in PIANO mode provide deeper bulk heating, stimulating fibroblast activity and vascular remodeling.^34^ FRAC3 mode uses fractional photothermolysis to improve skin texture with minimal downtime by creating localized thermal injuries that activate fibroblasts and HSPs.^35,36^ Q-switched Nd:YAG lasers deliver melanin-targeting pulses in the nanosecond range, effectively treating pigmentation without significantly affecting deeper dermal structures.^37^ Together, these modalities address the 3 principal dimensions of facial aging: mechanical descent, volumetric loss, and dermal degeneration. Thermal stimulation activates key molecular pathways (eg, HSP70, mTOR/p70S6K), enhancing fibroblast proliferation and ECM production.^38,39^ Simultaneously, it intensifies the paracrine activity of ADSCs, further amplifying tissue regeneration.^40,41^ Experimental models have shown improved adipocyte retention, increased vascular density, and superior integration when fat grafts are combined with low-intensity laser treatments.^42^ These mechanistic insights support the clinical observation that thermal stimulation through lasers augments the regenerative effects of fat grafting, particularly through enhanced HSP70 expression, mTOR pathway signaling, and ADSC activation.

Our findings remained robust after regression adjustment, supporting the biological plausibility of the observed synergistic effects. The dermatologist consistently identified greater improvements in skin texture and pigmentation compared with the plastic surgeon, who focused primarily on lifting effects. This divergence likely reflects the different perspectives of the 2 specialties: dermatologists are more attuned to subtle changes in skin quality parameters, whereas plastic surgeons primarily focus on lifting and contour. As a result, improvements in pigmentation and fine texture may be underestimated when assessed only from a surgical standpoint. These differences underline the importance of multidisciplinary evaluation in multimodal facial rejuvenation studies, since relying on a single specialty perspective risks overlooking clinically meaningful domains of improvement. Objective outcome measures, such as imaging or histology, should complement, rather than replace, these complementary clinical viewpoints.

Clinically, nonabsorbable thread lifting provided reliable tissue repositioning, whereas multimodal laser therapy yielded the most pronounced improvements in skin texture and patient satisfaction. The addition of autologous fat grafting was associated with a modest incremental benefit in fine wrinkle scores. Combining all 3 modalities produced the broadest spectrum of rejuvenation effects observed in this cohort. Importantly, although mechanisms such as the regenerative effects of fat or histologic remodeling with laser are supported by previous literature, these were not directly evaluated in our study. Future investigations incorporating objective volumetric analyses and histologic endpoints will be necessary to validate and expand upon these findings.

### Limitations

This study has several limitations. First, the retrospective design inherently limits causal inference and introduces potential selection bias, particularly because treatment allocation was patient driven rather than randomized. Second, although outcomes were evaluated by blinded assessors using validated Likert-scale metrics, we did not include histological or objective imaging confirmation (eg, ultrasound or biopsy), which restricts mechanistic validation. Finally, the cohort consisted exclusively of female patients, with skin phototypes predominantly II to IV. This demographic homogeneity may limit generalizability to other regions with different sex or phototype distributions. Prospective research in this domain remains limited, in part because of the reluctance of institutional review boards to authorize such protocols without robust, independent long-term evidence. Securing university-based funding is similarly challenging; although industry sponsorship is often accessible, it may raise concerns regarding the perceived independence of results. Generating high-quality, nonindustry-sponsored studies could facilitate future approval and funding of prospective multicenter trials. Future prospective, randomized, multicenter studies incorporating histological and advanced imaging analyses must comprehensively substantiate and extend these findings.

## CONCLUSIONS

The tri-modal strategy integrating nonabsorbable thread lifting, autologous fat grafting, and multimodal nonablative laser therapy provided superior aesthetic outcomes compared with thread lifting alone. Laser therapy contributed the greatest improvements in skin texture and overall satisfaction, whereas the addition of fat grafting further enhanced wrinkle reduction and overall scores. Collectively, the combination protocol achieved the most comprehensive rejuvenation outcomes in this cohort, underscoring the value of an integrative approach in nonsurgical facial rejuvenation.
